# Chicago Pathological Society, Meeting of September 10

**Published:** 1883-10

**Authors:** J. E. Harper

**Affiliations:** Secretary pro tem.


### ARTICLE X.

CHICAGO PATHOLOGICAL SOCIETY. Regular Meeting, September 10, 1883.

The Society was called to order by the President, Dr. Angear.

Dr. J. E. Harper was elected secretary pro tem. The reading of the minutes was dispensed with.

The Society first listened to the reading of a paper by Dr. Charles Warrington Earle, entitled, "Cephalhæmatoma of the New-Born."

This condition, consisting in a soft tumor of the bone, is more common than usually supposed. It is generally formed on the right parietal bone. Prof. Byford, and one or two others, have reported a double hæmotoma. The seat of the tumor is between the bones and the periosteum, and is caused by the rupture of a vessel.

Ætiology.—We are taught that these cases are due to the pressure of a rigid os upon the scalp. It is liable to be confounded by—1st, caput succedaneum, a condition of œdema; 2d, hernia cerebri; 3d, vascular tumor; 4th, protrusion of the cerebral mass through an opening made by syphilis. The treatment consists in a judicious non-interference.

The paper being before the Society for discussion and remarks, Dr. Lydston stated that he had seen two cases, both over the left parietal bone.

In syphilitic children, meningeal hæmorrhage often occurs after a few days, and the child succumbs.

Dr. Earle then read a second paper, entitled, "Disease of the Spleen, and Report of a Fatal Case."

June 28, Mrs. C. had an ordinary attack of indigestion; five days after he saw her again, and found temperature 101°, pulse 80, abdomen distended, and the lady in considerable pain. June 30 she began to fail, and pulse was more rapid; July 2, rapidly failing, with all symptoms of purpura hæmorrhagica.

Autopsy.—Spleen enlarged, weighing 19 ounces, showing suppuration; microscopic examination showed fatty degeneration of the arteries.

Diagnosis.—Pernicious progressive anæmia resulting from a peculiar sepsis following sewer-gas poisoning. Dr. Earle regarded it as a rare case of splenic disease.

Dr. Angear would like to know whether the microscopist was an experienced one, and if his statement that the arterial coats had undergone fatty degeneration could be relied upon? This being the case, hæmorrhage resulted and was followed by peritonitis which caused death.

Dr. Mergler had seen the post-mortems of four or five cases of pernicious anæmia, and in all the abdominal organs were very pale. In this case Dr. Earle stated that there was no discoloration noticeable.

Dr. Lydston recognized some similarity to cases of typhus fever as far as the splenic condition, ecchymoses, etc.

Dr. Earle asked if any one present had seen an autopsy in a case of leucæmia ?

The society then proceeded to discuss the subject of "Sponge Grafting," so called. Dr. Angear reported a case of a lady about 40 years of age who had an ulcer on the lower extremity, of six years standing, and who had not menstruated during this time. At each period hæmorrhage occurred from this ulcer. After trying the usual methods without success, in desperation, tried sponge grafting. One week after applying sponge the entire ulcer had healed ; but in about six weeks after the ulcer reopened, and all the new skin, with the exception of a few islets, sloughed off.

Dr. Newton reported a case in which six grafts were used, and had a good result.

Dr. Tagert thought it advisable to cover the entire surface with the sponge. Dr. Newton called attention to Dr. Hamilton's later method of using sponge in small pieces, in preference to large ones. Dr. Angear thought the sponge serves only as a protection to the delicate blood vessels and cells until the new tissue is formed, and hence the term "sponge grafting" is [illegible] leading.

Dr. Patton thought the results obtained from both skin and sponge grafting depends upon the establishment of new and independent foci for the production of plastic lymph.

The society, on motion, ajourned.

J. E. HARPER,
*Secretary pro tem.*

